# Third molars: To extract or not to extract?

**DOI:** 10.1590/2176-9451.20.4.017-018.edt

**Published:** 2015

**Authors:** David Normando

**Affiliations:** 1 Adjunct Professor, School of Dentistry, Universidade Federal do Pará (UFPA). Head of the post-graduation program in Dentistry, Universidade Federal do Pará and Specialization Course in Orthodontics, ABO-Pará.

Good decisions come from experience, and experience comes from bad decisions. Albert Einstein

Third molar extraction is one of the most frequent procedures in oral surgery. Ten million teeth are extracted from approximately five million individuals every year in the United States.^1^ The reported reasons for third molar removal include the risk of impaction associated with caries, pericoronitis, periodontal defects in the distal surface of second molars, odontogenic cysts and dental crowding. A prospective study^2^ showed that general dentists recommend extraction of third molars in 59% of patients, mainly to prevent future problems or because a third molar had an unfavorable orientation or was unlikely to erupt. However, the power to predict third molar eruption is low, and impacted third molars that remain static, with no changes in position or angulation over time, are rare.

The ideal moment to determine whether or not to remove third molars is also under debate, since impaction prediction has not been scientifically proven. Moreover, it is a daunting task to predict this biological condition with any degree of reliability. Systematic reviews report that there is no evidence to support or refute prophylactic removal of asymptomatic impacted third molars, even in adults.^3^^,^ 4 These systematic reviews contraindicate the prophylactic removal of third molars in order to prevent late lower anterior crowding. However, in comparing the opinion of orthodontists and oral and maxillofacial surgeons, it became clear that the latter indicate prophylactic removal of third molars to prevent crowding more often than the former.^5^

Whenever indicating extraction of third molars, dentists should have a justifiable reason, one that takes into account future treatment planning from an orthodontic, surgical, periodontal and/or prosthetic point of view. At the same time, a cost/benefit analysis should be carried out to justify the prophylactic removal of third molars, which should only be indicated with the purpose of preventing cases that involve pathological processes, such as root resorption or caries in second molars, cysts and pericoronitis. 

Furthermore, dentists and patients must take into account that surgical complications after third molar removal are common. The prevalence of seeking postsurgical emergency appointments is around 10%.^6^^,^ 7 The reasons are severe pain, swelling, bleeding, alveolar osteitis, abscesses, dehiscences, sequestra paresthesia, hematoma, and trismus. Although uncommon, there are hundreds of reports on jaw fracture after third molars surgeries published in the literature.^8^ These fractures predominantly occur in patients who are older than 25 years. Thus, it seems reasonable to believe that postponing the extraction of third molars can increase the risk of mandibular fracture. 

On the other hand, third molars can be used to replace a first or second molar previously extracted. Also, because stem cells can be derived from healthy human third molars,^9^^,^ 10 they represent an easily accessible source which opens a range of new possibilities for regenerative medicine.

For orthodontic patients, the decision whether or not to remove third molars could be postponed until the end of orthodontic treatment, except for situations in which the removal of a third molar is mandatory since the beginning of treatment. A follow-up evaluation of third molar position during treatment can contribute to a more realistic decision prognosis of these teeth. If orthodontic treatment is complete before the final positioning of these teeth is achieved, the patient should be reassessed by clinical examination and periodic radiographic. In general, not deciding is the best decision for such cases.

David Normando - editor-in-chief (davidnormando@hotmail.com)
